# Atypical optic neuritis – a case with a new surprise every visit

**DOI:** 10.3205/oc000138

**Published:** 2020-02-27

**Authors:** Kirandeep Kaur, Bharat Gurnani, Nirmala Devy

**Affiliations:** 1 Aravind Eye Hospital, Pondicherry, India; 2 Aravind Eye Hospital, Chennai, India

**Keywords:** neuritis, demyelination, tuberculosis

## Abstract

Demyelination is the most common cause of optic neuritis. Typical optic neuritis needs intravenous steroids followed by tapering dose oral steroids. Atypical optic neuritis entails clinical manifestations that deviate from the classic pattern of features. Atypical ON can have devastating visual results if not treated in a timely fashion. Thus, it is critical that cases of atypical optic neuritis are recognized early to initiate proper treatment and preserve vision. Our case presented as an atypical case of optic neuritis. The patient had painless onset and was not responding to steroids, but presentation in the other eye and systemic administration of antivirals helped in management of this case.

## Introduction

Optic neuritis refers to inflammation of the optic nerve [1]. As per western literature, Optic neuritis occurs in about 50% of patients with multiple sclerosis and it is the presenting feature in 20% of cases [2]. The incidence of acute optic neuritis in general population has been reported as 1/100,000 [3]. A female-to-male ratio of 5:1 is reported [4]. The young age group (20–40 years) is most commonly affected [5]. Demyelination has been reported as the most common cause [6]. There is no need of laboratory investigations, i.e. ESR or ANA. The need for MRI to assess multiple sclerosis risk has been highlighted.

## Case description

### History

A 56-year-old male patient, driver by occupation, came to our hospital for a medical fitness certificate. He reported left eye redness, which he had suffered from for 6 months on and off, with no associated complaints of irritation, pain, or photophobia. Uncorrected visual acuity (UCVA) in the right eye was 6/9 and 6/36 in the left eye, with a best corrected visual acuity (BCVA) of 6/6 in the right eye and 6/9 in the left eye.

### Examination

**Figure 1:**
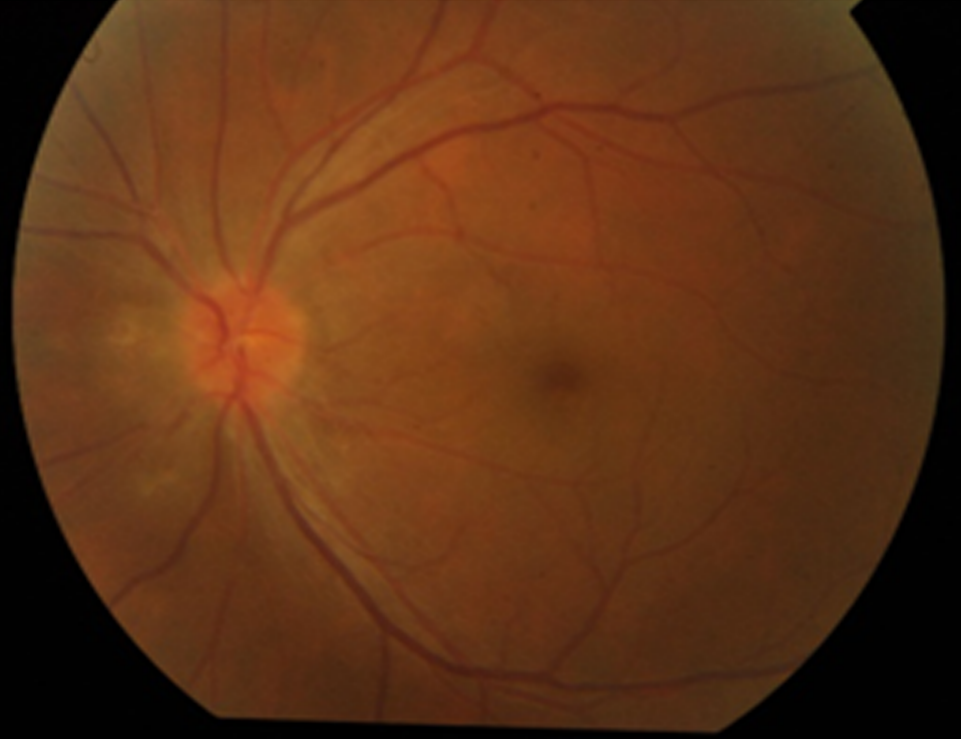
Fundus image of left eye showing blurred disc margins with choroidal folds

Anterior segment examination of the right eye was normal with brisk reacting pupil and sluggish consensual reaction. The examination of the left eye was normal, except for a grade 1 relative afferent pupillary defect (G1 RAPD). Posterior segment examination was normal in the right eye, but examination of the left eye showed blurred disc margins (Figure 1 (Fig. 1)) with choroidal folds.

### Ancillary testing

**Figure 2:**
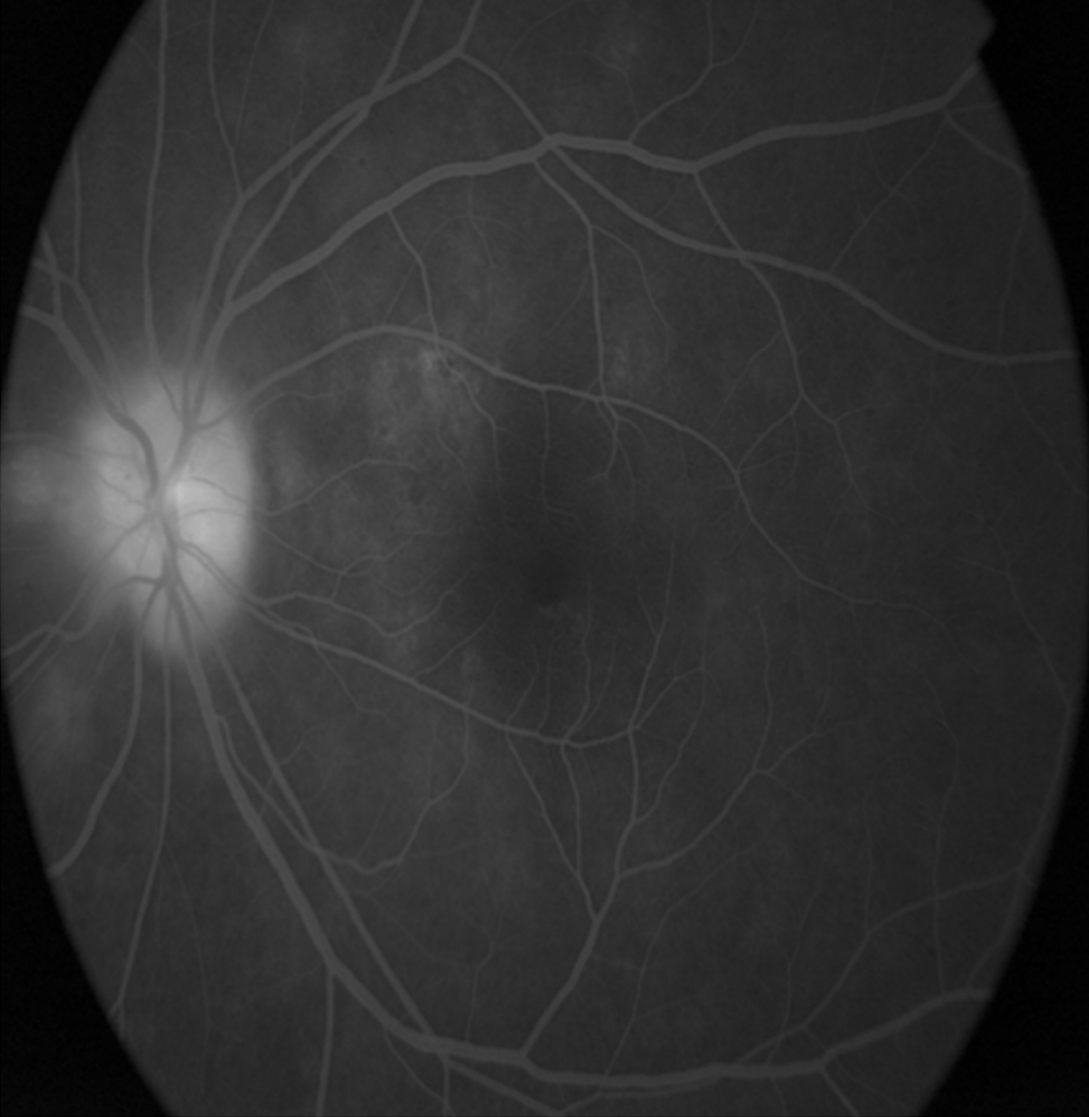
Fundus flourescein angiography of left eye depicting disc leakage

**Figure 3:**
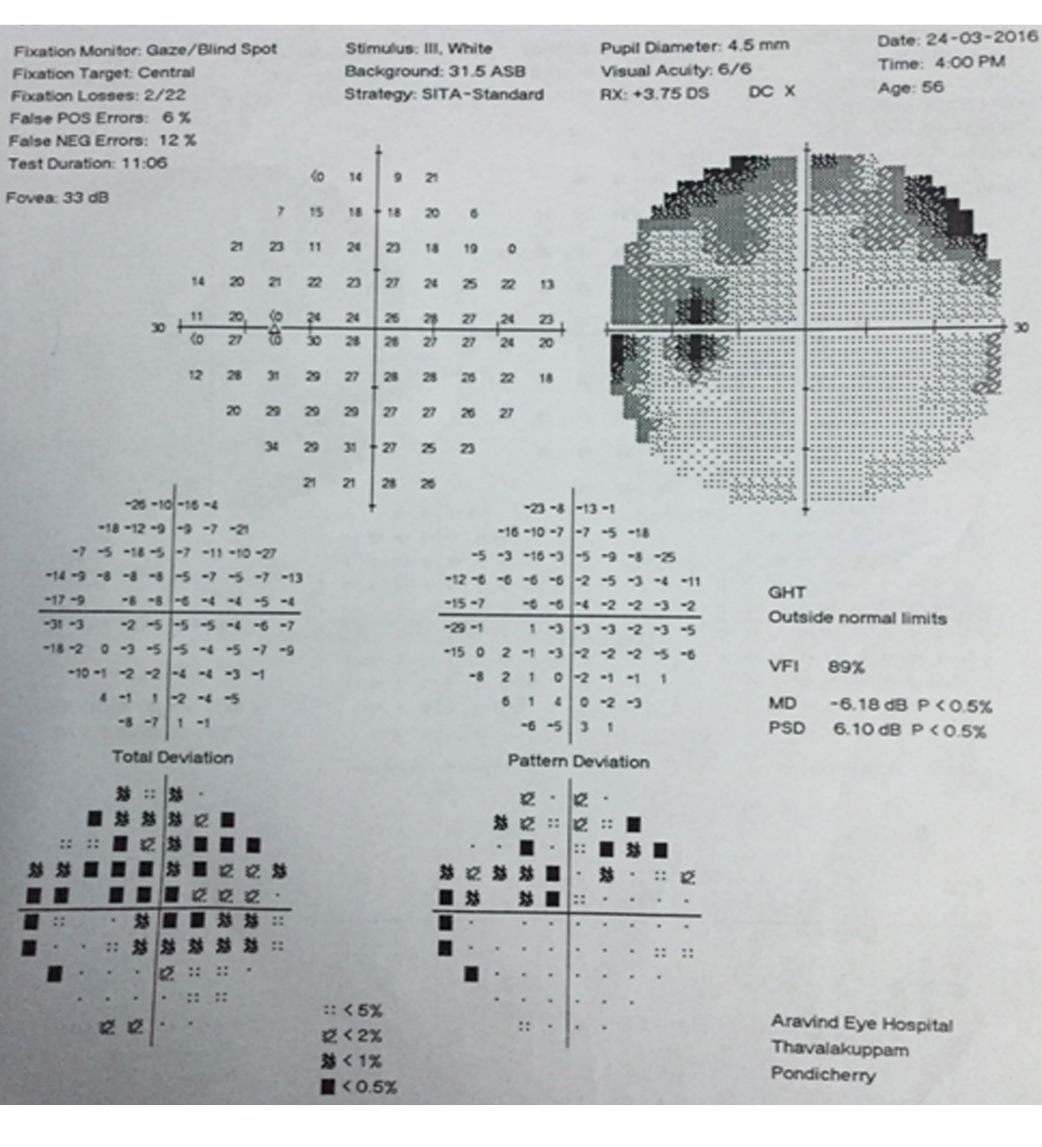
Humphrey field analysis 30-2 showing left superior arcuate scotoma

To identify the causative factor, we advised the patient to undergo color vision, blood investigations, chest X-ray, Mantoux test, and MRI of the brain, all of which turned out to be normal. Disc leakage was seen on fluorescein angiography (FFA) (Figure 2 (Fig. 2)), and Humphrey field analyzer (HFA) 30-2 showed superior arcuate scotoma (Figure 3 (Fig. 3)). Visual evoked potential (VEP) showed reduced amplitude and prolonged P100 latency.

### Treatment

The final diagnosis of atypical optic neuritis was made, and pulse therapy with intravenous methylprednisolone 1 g/day for 3 days was started, followed by oral steroids and vitamin supplements. On subsequent visits, we stopped steroids as per Optic Neuritis Treatment Trial (ONTT), though the left eye disc margins blurring persisted. Repeat HFA 30-2 was not reliable.

### Review visit

**Figure 4:**
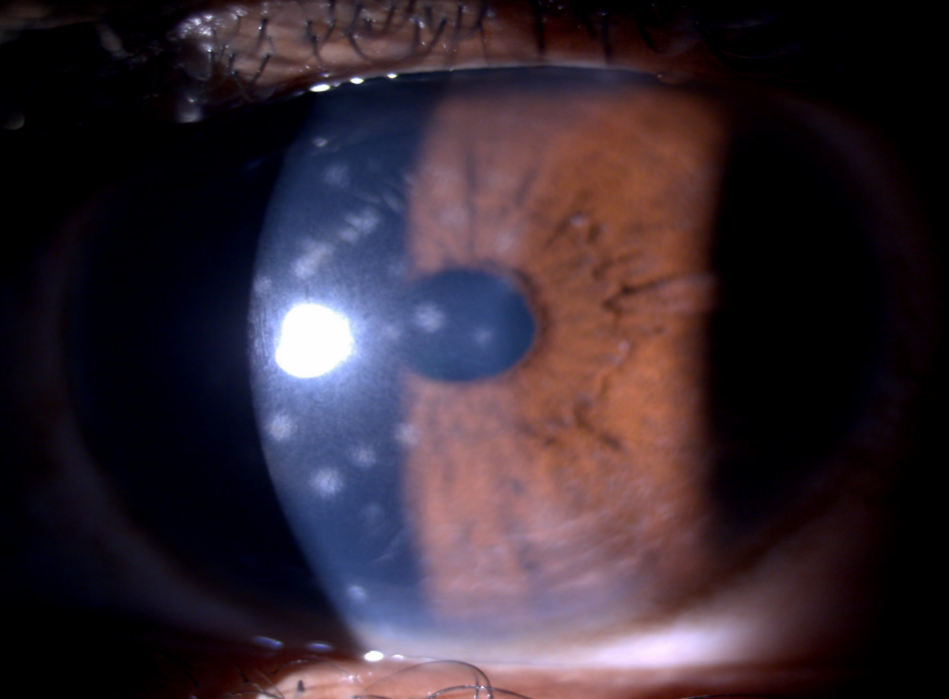
Slit-lamp photo showing circular subepithelial infiltrates in the right eye

One month later, the patient presented with chief complaints of painful decreased vision in the right eye associated with photophobia and redness. BCVA in the right eye was noted to be 6/18 and 6/6 in the left eye. Anterior segment examination in the right eye revealed circumcorneal congestion, subepithelial keratitis with 0.5+ cells in the anterior chamber (Figure 4 (Fig. 4)). In the left eye, there was persistence of G1 RAPD with blurred disc margins. A diagnosis of right-eye viral keratouveitis and left-eye atypical optic neuritis was made. The patient was started on oral tablets acyclovir 800 mg twice a day for 2 weeks with gancyclovir eye ointment, prednisolone with moxifloxacin eye drops for topical application. Two weeks later, the patient presented with BCVA of 6/6 in both eyes. and surprisingly, fundus evaluation was normal with resolved disc edema. This was a case of atypical optic neuritis with viral aetiology, which responded well to antiviral drug therapy.

## Discussion

Optic neuritis refers to inflammation of the optic nerve [1]. Optic neuritis occurs in about 50% of patients with multiple sclerosis, and it is the presenting feature in 20% of cases [2]. A female-to-male ratio of 5:1 has been reported in earlier studies [4]. The young age group (20–40 years) is the most commonly affected [5]. Demyelination has been reported as the most common cause in western literature [6]. There is no need of laboratory investigations, i.e. ESR or ANA. MRI has been highlighted to assess multiple sclerosis risk. Classic demyelinating optic neuritis is associated with multiple sclerosis and carries a good prognosis for visual recovery. Atypical optic neuritis has manifestations that deviate from the classic features. Atypical features include lack of pain, simultaneous or near-simultaneous onset, lack of response to or relapse upon tapering from corticosteroids, or optic nerve head or peripapillary hemorrhages [7]. The “atypical” causes of optic neuritis include neuromyelitis optica (NMO), autoimmune optic neuropathy, chronic relapsing inflammatory optic neuropathy (CRION), idiopathic recurrent neuroretinitis (NR), and optic neuropathy associated with systemic diseases. Atypical optic neuritis can have devastating visual results if not treated appropriately in a timely fashion. Thus, it is critical that cases of atypical optic neuritis are recognized early to initiate proper treatment and preserve vision as much possible [8]. Our case presented as an atypical case of optic neuritis. The patient had painless onset and was not responding to steroids, but presentation in the other eye and systemic administration of antivirals helped in management of this case.

## Conclusions

1. Optic neuritis can have varied presentation.
2. The clinical presentation and response to treatment outweighed the investigations in this patient.

## Notes

### Competing interests

The authors declare that they have no competing interests.

### Declaration of patient consent

The authors certify that they have obtained appropriate consent from the patient. In the consent form, the patient has given his/her consent for his/her images and other clinical information to be published in the journal. 

### Study location

The work was carried out at Aravind Eye Hospital and Post-Graduate Institute of Ophthalmology, Thavalakuppam, Cuddalore Main Road, Pondicherry 605007, India.
